# Family planning utilization and associated factors among postpartum women in Addis Ababa, Ethiopia, 2018

**DOI:** 10.1371/journal.pone.0245123

**Published:** 2021-01-22

**Authors:** Lema Tafa, Yoseph Worku

**Affiliations:** 1 Family Health Core Process, Nifas-Silk Lafto Sub-city Health Office, Addis Ababa, Ethiopia; 2 Department of Public Health, St. Paul’s Hospital Millennium Medical College, Addis Ababa, Ethiopia; Queen's University at Kingston, CANADA

## Abstract

**Background:**

Pregnancies that occur in the first year after birth can result in adverse outcomes for the mothers and their babies. Postpartum family planning (PPFP) can save lives of many mothers and children. Only few data are available about the magnitude of PPFP use and its determinants in Addis Ababa, Ethiopia.

**Objective:**

To assess PPFP utilization and associated factors in Addis Ababa, Ethiopia.

**Method:**

A facility-based cross-sectional study was conducted from April to June 2018. A total of 625 women were enrolled in the study. Statistical Package for the Social Sciences (SPSS) software was used to analyze the data. Binary logistic regression model with adjusted odd ratio (AOR) and 95% confidence interval (CI) was used to identify the factors associated with PPFP use. A p-value less than 0.05 was considered as significant.

**Result:**

The magnitude of PPFP utilization in Addis Ababa was 71.8%. Previous family planning (FP) information (AOR = 13.2; 95% CI: (1.96, 88.07)), FP information from health facility visit (AOR = 2.23; 95% CI: (1.45, 3.43)), antenatal care (AOR = 4.96; 95% CI: (1.58, 15.64)), counseling on FP at postnatal care (AOR = 1.97; 95% CI: (1.27, 3.05)), menses resumption after birth (AOR = 1.75; 95% CI: (1.11, 2.76)), and commencing sexual activity after birth (AOR = 9.34; 95% CI: (5.39, 16.17)) were the factors associated with PPFP use.

**Conclusion and recommendation:**

Though the magnitude of PPFP use is encouraging, still three out of the ten postpartum women did not use PPFP. The determinants of PPFP use were having FP information, having FP information from health facility visit, antenatal care, counseling about FP during postnatal care, menses resumption after birth, and commencing sexual activity after birth. The health system in the City and the healthcare providers should strive to reach every woman who is not accessing the PPFP services and antenatal care services, and improve counseling services on PPFP during delivery and postnatal care services.

## Introduction

Family planning (FP) has several benefits for women, children and families; and it is considered as a human right. But, millions of women in the world are lacking safe and effective FP methods despite their need. According to 2015 United Nation’s report, globally, 12% of married or in-union women had an unmet FP need. The FP use was very low in Africa at 33% and the unmet FP need was high in sub-Saharan Africa (SSA) at 24% [1]. Eight out of ten (82%) unintended pregnancies occur among women with unmet FP need and unintended pregnancies lead to high levels of unplanned births, unsafe abortions, and maternal death [2].

Unintended pregnancy occurring in early postpartum period is associated with more common and worse adverse outcomes, but the magnitude is still unacceptably high. Worldwide, about 80 million unintended pregnancies occur due to low FP use during the postpartum period [3]. In SSA, 40% of unmet need of FP occurs among postpartum women [4]. As two thirds of maternal and neonatal mortalities occur during the postpartum period [5], proper utilization of postpartum family planning (PPFP) in the 12 months following childbirth can considerably reduce those adverse outcomes [6,7]. World Health Organization recommends maternal health care services and routine immunization services should be key service delivery points for provision of PPFP information and services [8]. However, reports and studies reveal that in SSA postpartum women do not get adequate FP information or services from these service delivery points [9,10].

Ethiopia has one of the highest number of maternal mortalities in the world, with high FP unmet need resulting around 38% of pregnancies being unplanned (85 per 1,000 women) and 13% of unintended pregnancies resulting in induced abortion in 2014 [11]. Ethiopian Demographic and Health Survey (DHS) report shows that the demand for FP among married women increased over time rising from 45% in 2000 to 58% in 2016 and the need met for FP has also increased over the same period from 8% in 2000 to 36% in 2016. Though the FP use increased through the years, unmet need for FP still remained high which is 22% and 11% at national and Addis Ababa level, respectively in 2016 [12]. In Ethiopia, there is high unmet need among postpartum women and nearly half (47%) of all pregnancies occur within a short birth interval of less than 24 months after the preceding birth [13].

Though the magnitudes of unmet FP needs have been well-assessed overall, there is a scarcity of evidence about PPFP use and its determinants in Addis Ababa, Ethiopia. Therefore, this study aimed to assess the utilization of FP and associated factors among postpartum women attending public health facilities for child immunization service in Addis Ababa, Ethiopia, in 2018.

## Methods

Sante Medical College Institutional Review Board and Addis Ababa City Administration Health Bureau Ethical Clearance Committee approved the study. Informed written consent was obtained from each of the participants.

### Study setting

This study was conducted in Addis Ababa City Administration, Ethiopia. Addis Ababa is the capital city of Ethiopia and seat of the African Union. The City is comprised of ten sub-cities and 116 woredas (equivalent to districts) with an estimated population of 3,434,0000 (male 1,625,000 and female 1,809,000) in 2017 [14]. There are 96 public health centers in the city that provide maternal and child healthcare services, including PPFP and immunization services.

### Study design and period

We conducted a facility-based cross-sectional study from April to June 2018.

### Study population and eligibility criteria

Postpartum women who were attending selected health centers for child immunization services and who had given birth during the 12 month period prior to the study were enrolled. Any woman who had some form of apparent illness or discomfort was excluded from the study.

### Study variables

The dependent variable of the study was PPFP use and the independent variables were age, educational status, marital status, occupation, antenatal care, postnatal care, place of delivery, previous FP information, FP information from health facility visit, number of living children, birth interval, knowledge on FP and attitude towards FP.

### Sample size determination

Sample size was estimated for each specific objective of the study using Epi Info Version 7.2.1.0 STATCACL, and we took the largest among all estimations. For the first three objectives (levels of knowledge, attitude, and use of PPFP), we used the single population proportion formula (Annex 1) and for the last specific objective (determining the associated factors), we used the two-population proportion formula (Annex 2). The final sample size, assuming a non-response rate of 10% and design factor of 1.5, was 633 study participants.

### Sampling procedures

The study participants were recruited using multistage sampling technique. First, we grouped the health centers in their respective sub-city. Then, we randomly selected one health center from the health centers in each sub-city. A total of ten health centers were selected from the ten sub-cities. Proportional number of study participants were allocated for each health center based on the average number of postpartum women who attended the immunization clinics over the three months prior to the study. From each health center, the study participants were recruited using systematic random sampling technique. Having considered the estimated number of women to attend every health center during the data collection period (N = 3,388) and the estimated sample size (n = 633), the sampling interval was calculated (K = 5). i.e. K = N/n = 3,388/633 = 5.4. The first participant was selected randomly by lottery method from 1 to 5. Then, every 5^th^ eligible woman coming to the immunization clinic was enrolled until calculated sample size was achieved.

### Data collection instrument and technique

Data was collected using an interviewer-administered questionnaire which was adopted mainly from the EDHS questionnaire and other similar studies [13,16,21]. The questionnaire was prepared first in English and then translated to Amharic and back to English by two language experts. The questionnaire had six parts: *socio-demographic characteristics*, *reproductive history*, *PPFP knowledge*, *attitude towards PPFP*, *PPFP use*, *and list of factors that could be associated with PPFP*. Five data collectors (diploma holding nurses) and two supervisors (degree holding nurses) were recruited for the interviews.

### Data quality assurance

To ensure the quality of the data, the data collectors and the supervisors were provided two-days of training on the objective of the study, how to administer the questionnaire, how to identify errors and how to correct identified errors. The questionnaire was pre-tested on 32 postpartum women at health centres that were not selected for the study and revised according to the feedbacks. Regular supervision was conducted by the principal investigator and recruited supervisors during the data collection. Questionnaires with incomplete, inconsistent, and inaccurate data were identified promptly and corrected appropriately.

### Data analysis

Data was analyzed using IBM Statistical Package for the Social Science (SPSS) Statistics for Windows, Version 20.0. Armonk, NY: IBM Corp. Frequency distributions, percentages, mean, and standard deviation (SD) were used to describe the data. Mean and SD of the knowledge and attitude question scores were calculated. For the binary logistic regression model, key assumptions and fit of the model were assessed. Presence of influential values was examined by visualizing the Cook’s distance values and a value of greater than one was used to identify the influential variables. Multicollinearity was checked using the correlation matrix and a correlation coefficient > 0.8 was used to pick variables with high correlation. The overall goodness-of-fit of the model was assessed using Hosmer and Lemeshow Test and p-value > 0.05 was used to conclude the model fitness. First, to assess the relationship between each of the independent variables and the dependent variable (PPFP use), simple binary logistic regression was used and the p-values were determined. To control confounders, multiple binary logistic regression modelling was used and all the independent variables with p-value ≤ 0.2 in the simple binary logistic regression were considered for the final model. Adjusted odds ratios (AOR) with 95% confidence interval (CI) were used to quantify the magnitude of the associations between the independent variables and the PPFP use; p-value < 0.05 was considered significant.

### Operational definition

Knowledge of PPFP was assessed based on a set of seven questions that were scored either ‘1’ (correct) or ‘0’ (incorrect). Thus, the minimum and maximum possible total knowledge scores for each participant were ‘0’ and ‘7’, respectively. Finally, knowledge was categorized into good and poor by using the mean knowledge score as a cutoff point [15]. Attitude towards PPFP was assessed by a set of ten questions that were rated based on the five-points Likert scale ([‘5’ = strongly agree], [‘4’ = agree], [3 = neutral], [2 = disagree], and [1 = strongly disagree]). Thus, the minimum and maximum possible total attitude scores for each participant were ‘10’ and ‘50’, respectively. Finally, attitude was categorized into favourable and unfavourable using the mean attitude score as a cutoff point [15]. PPFP use was defined as the use of FP during the 12 months period after giving birth and was categorized into PPFP user and non-user based on the current use of any form of FP between giving birth and the date of the interview.

## Results

### Characteristics of study participants

A total of 625 eligible postpartum women were enrolled in the study with response rate of 98.7%. The mean age of the respondents was 27.6 years (SD = 4.7 years). Two hundred sixty-two (41.9%) respondents were in the 25–29 age range. A majority of the participants (92.8%) were married and 321 (51.3%) were housewives. One hundred seventy-two (27.5%) and 298 (47.7%) participants had attended secondary and tertiary level education, respectively (Table 1).

**Table 1:** Socio-demographic characteristics of postpartum women in selected public health facilities, in Addis Ababa, Ethiopia, 2018 (n = 625).

Variables	Frequency	Percentage
**Age (years)**		
15–19	7	1.1
20–24	157	25.1
25–29	262	41.9
30–34	133	21.3
≥35	66	10.6
**Educational level**		
No formal education	58	9.3
Primary level (Grades 1–8)	97	15.5
Secondary level (Grades 9–12)	172	27.5
Tertiary level (Diploma and above)	298	47.7
**Religion**		
Orthodox	395	63.2
Muslim	141	22.5
Protestant	88	14.1
Catholic	1	0.2
**Marital status**		
Married	580	92.8
Single	25	4
Divorced	17	2.7
Widowed	3	0.5
**Occupation**		
Self-employed	168	26.9
Government employee	136	21.8
House wife	321	51.3

### Reproductive history and healthcare service utilization

The majority of the women (96.5%) had at least one ANC visit and 436 (72.3%) had four or more focused ANC visits for their most recent birth. Nearly all the participants (99.4%) had delivered their youngest child at a health facility. The majority of the participants (96.8%) visited a health facility during the postpartum period. Postpartum family planning counseling was provided during ANC, delivery and PNC for 449 (74.5%), 301 (48.5%) and 302 (48.3%) women, respectively. The mean number of children among study participants was 1.83 (SD = 0.89). Among the 363 mothers who have more than one child, the median time between the recent and prior birth was 24 months and 123 (33.9%) of the women had birth spacing less than 24 months (Table 2).

**Table 2:** Health service characteristics of the study participants in selected public health facilities in Addis Ababa, Ethiopia, 2018 (n = 625).

Variables	Frequency	Percentage (%)
**ANC visit (n = 625)**		
Yes	603	96.5
No	22	3.5
**Number of ANC visits (n = 603)**		
One	34	5.6
Two	39	6.5
Three	94	15.6
Four and above	436	72.3
**Counseled for PPFP during ANC visit (n = 603)**		
Yes	449	74.5
No	154	25.5
**Place of delivery for recent child (n = 625)**		
Hospital	287	45.9
Health center	334	53.4
Home	4	0.6
**Counseled for PPFP during delivery (n = 621)**		
Yes	301	48.5
No	320	51.5
**Any visit of HF after giving recent birth (n = 625)**		
Yes	605	96.8
No	20	3.2
**Counseled for FP during PNC (n = 625)**		
Yes	302	48.3
No	323	51.8
**Number of living children (n = 625)**		
One	267	42.5
Two	234	37.4
Three	85	13.6
Four	39	6.3
**Birth interval before recent delivery (n = 625)**		
First birth	262	41.9
< 2 years	123	19.7
2–3 years	103	16.5
>3 years	137	21.9

ANC: Antenatal care, PPFP: Postpartum family planning, HF: Health facility, FP: Family planning, PNC: Postnatal care.

### Knowledge and attitude towards FP

Based on the seven knowledge questions, the mean of the correctly answered questions was 3.32 (SD = 1.53). Two hundred thirty-two (37.1%) participants had good knowledge of FP. A majority of the respondents (88.8%) knew that fertility resumes once contraceptive is stopped; around a third (34.7%) of the participants knew contraceptive use helps to limit the number of children a woman can have and only a fifth (19.4%) knew using FP helps to prevent maternal morbidities and mortalities. Among the different FP methods, a majority (57.0%) of the respondents knew about injectables and only 1.6% of the participants knew about the withdrawal method.

Based on the ten attitude questions, the mean score of the respondents was 34.49 (SD = 3.81). Half of the respondents (50.9%) had a favorable attitude towards FP. One hundred seventy-six (28.2%) participants did not agree that PPFP use helps a woman to regain her strength before her next baby. Eighty-six (13.8%) participants did not agree that men should share the responsibility of PPFP use (Table 3).

**Table 3:** Attitude of postpartum women towards FP in selected public health facilities in Addis Ababa, Ethiopia, 2018 (n = 625).

Variables	Strongly agree n (%)	Agree n (%)	Neutral n (%)	Disagree n (%)	Strongly disagree n (%)
PPFP is good for mother and child health	210 (33.6)	370 (59.2)	26 (4.2)	13 (2.1)	6 (1.0)
Discussing PPFP use with partner is good	211 (33.8)	380 (60.7)	20 (3.2)	10 (1.6)	4 (0.6)
Men should share the responsibility of PPFP use	170 (27.2)	369 (59.0)	40 (6.4)	36 (5.8)	10 (1.6)
PPFP helps a mother to regain strength before her next baby	119 (19.0)	330 (52.8)	106 (17.0)	60 (9.6)	10 (1.6)
Women need to encourage their friends to use PPFP	157 (25.1)	337 (53.9)	60 (9.6)	64 (10.2)	7 (1.1)
Unmarried women can use FP methods	76 (12.5)	237 (37.9)	76 (12.1)	188 (30.1)	48 (7.7)
Using contraceptive could not affect cultures	31 (5.0)	90 (14.4)	146 (23.4)	319 (51.0)	39 (6.2)
Religion does not forbid contraceptive use	58 (9.3)	173 (27.7)	99 (15.8)	225 (36.0)	70 (11.2)
Using contraceptive cannot cause infertility	89 (14.2)	160 (25.6)	303 (48.5)	66 (10.6)	7 (1.1)
Husband should not decide if wife wants to use FP	196 (31.3)	328 (52.4)	18 (2.9)	72 (11.5)	11 (1.8)
Favorable Attitude	318		50.9%		
Unfavorable Attitude	307		49.1%		

PPFP: Postpartum family planning, FP: Family planning.

### PPFP utilization

Four hundred forty-nine of the participants (71.8%; 95% CI: (68.3, 75.2)) were using PPFP. The first widely used contraceptive methods were injectables (32.1%), followed by implants (28.7%). Majority of the PPFP users (81.7%) got the service from public health facilities. Six out of ten PPFP users (59.2%) started to use a contraceptive at six weeks postpartum (Table 4).

**Table 4:** PPFP use of the study population in selected public health facilities in Addis Ababa, Ethiopia, 2018.

Variables	Category	Frequency	Percent
**PPFP use (n = 625)**	Yes	449	71.8
	No	176	28.2
**Type of PPFP method (n = 449)**	Injection	144	32.1
	Implant	129	28.7
	Pill	95	21.3
	IUCD	74	16.5
	Male condom	7	1.4
**PPFP method/service delivery point (n = 449)**	Public health facility	367	81.7
	Private health facility	55	12.3
	NGOs	19	4.2
	Pharmacy	8	1.8
**Time of PPFP use (n = 449)**	Before 6 weeks	30	6.7
	At 6 weeks	266	59.2
	6 weeks-3 months	88	19.6
	After 3 months	65	14.5

PPFP: Postpartum family planning, IUCD: Intrauterine contraceptive device, FP: Family planning, NGO: Non-governmental organization.

### Factors associated with PPFP use

As per the diagnostics for the key assumptions and fit of the logistic regression model, no influential value was identified (all the Cook’s distance values were greater than one); no multicollinearity was picked up (correlation coefficients < 0.8); the model was good enough for the data (Hosmer and Lemeshow Test with p = 0.47). And, based on the final multiple binary logistic regression analysis, having FP information, having FP information from health facility visit, ANC attendance, counseling during PNC, menses resumption, and commencing sexual intercourse were all associated with PPFP use.

Women who had previous FP information were 13 times as likely to report PPFP use compared to those who did not have previous FP information (AOR = 13.2; 95% CI: (1.96, 88.07)). Women who got FP information during a health facility visit were two times as likely to report PPFP use, compared to those who did not get the FP information (AOR = 2.23; 95% CI: (1.45, 3.43)). Participants who had ANC follow up were nearly five times higher likely to use PPFP, compared to those who did not attend (AOR = 4.96; 95% CI: (1.58, 15.64)). Women who had counseling during PNC were almost two times as likely to report PPFP use compared to those who did not get counseling during PNC (AOR = 1.97; 95% CI: (1.27, 3.05)). Menses resumption and commencing sexual intercourse were also associated with PPFP use (Table 5).

**Table 5:** Factors associated with postpartum family planning use in selected public health facilities, in Addis Ababa, Ethiopia, 2018 (n = 625).

Variables	PPFP users (n = 449)	PPFP non-users (n = 176)	COR 95% CI	AOR 95% CI	P-value
**Previous FP information**					
Yes	447 (72.4%)	170 (27.6%)	7.88 (1.58, 39.46)	13.2 (1.96, 88.07)*	**0.008**
No	2 (25.0%)	6 (75.0%)	1.00	1.00	
**FP information from HF visit**					
Yes	251 (79.4%)	65 (20.6%)	2.17 (1.51, 3.09)	2.23 (1.45, 3.43)**	**0.001**
No	198 (64.1%)	111 (35.9%)	1.00	1.00	
**ANC attendance**					
Yes	443 (73.5%)	160 (26.5%)	7.38 (2.84, 19.19)	4.96 (1.58,15.64)*	**0.006**
No	6 (27.3%)	16 (72.7%)	1.00	1.00	
**Menses resumption**					
Yes	334 (79.3%)	87 (20.7%)	2.97 (2.07, 4.27)	1.75 (1.11,2.76)*	**0.016**
No	115 (56.8%)	89 (43.6%)	1.00	1.00	
**Sexual resumption**					
Yes	412 (81.3%)	95 (18.7%)	9.4 (6.06, 14.7)	9.34 (5.39, 16.17)*	**0.001**
No	37 (31.4)	81 (68.6%)	1.00	1.00	
**Counseled at PNC**					
Yes	237 (78.3%)	65 (21.5%)	1.93 (1.35, 2.75)	1.97 (1.27, 3.05)*	**0.003**
No	212 (65.6%)	111 (34.4%)	1.00	1.00	

PPFP: Postpartum family planning, COR: Crude odds ratio, AOR: Adjusted odds ratio, CI: Confidence interval, FP: Family planning, HF: Health facility, ANC: Antenatal care, PNC: Postnatal care

* implies significant association with p-value <0.05

** implies significant association with p-value <0.001

1.00 represents reference category.

## Discussion

Postpartum family planning is one of the high impact interventions in preventing maternal and newborn morbidities and mortalities [3]. Thus, we assessed the utilization of FP and associated factors among postpartum women in one of the SSA countries where there is high unmet need of FP with a scarcity of local data.

Family planning utilization in Addis Ababa among women in the 12 months postpartum period was encouragingly high– 71.8%. This finding is in line with studies conducted in Malawi (75.0%) and Nigeria (73.3%) [16,17], but the figure was higher than studies conducted in some parts of Ethiopia: Somali region, Gondar town, and Aksum town which reported 12.3%, 48.4% and 48.0%, respectively [15,18,19]. The variation may be due to socio-demographic differences, awareness towards PPFP use, and the types of study (facility versus community-based). On other hand, this study finding was lower than the South West Addis Ababa, in Kolfe-Keranio sub-city which reported 80.3% [20]. This may be due to differences in study design and study population. The Kolfe-Keranio study enrolled women up to 24 months postpartum and the study was community-based, whereas our study was focused on women who were in the first 12 months postpartum and the study was facility-based. Though the magnitude of PPFP utilization is encouraging, the possible risk of unintended or closely-spaced pregnancies in the City is high; it apprises the need for further efforts in addressing the gap.

Most of the postpartum women (98.7%) knew at least one FP method. This is almost similar with the 2016 Ethiopian national survey which reported 99.0% [12] and another study conducted in Gojjam which reported 98.0% [21]. But the figure is higher than those of Malawian and Nepalese studies which reported 94.3% and 90.8%, respectively [16,22]. These disparities might be due to differences in the awareness levels of the study participants and time gaps between the studies.

In the current study, only 37.1% of women had good knowledge towards PPFP. This finding is higher than a study conducted in Eastern Ethiopia, Somali region, which used the same study tool and reported 31.8% [15]. This variation might be due to socio-demographic differences. Only half (50.9%) of the post-partum women had a favorable attitude towards PPFP. This finding is comparable to the study finding of Somali region which reported 51.0% [15]. The gaps in the basic knowledge and attitude aspects of PPFP among the study participants imply that there is still a need to further work on health education and promotion endeavors.

Postpartum family planning use was significantly associated with having previous FP information, FP information from health facility visit, ANC follow up, FP counseling during PNC, menses resumption, and commencing sexual intercourse. Women who had ANC follow up during pregnancy were 4.96 times as likely to use PPFP, compared to those who did not have ANC follow up. This finding was in concordance with a study conducted in Kebribeyah town, Eastern Ethiopia [15]. This implies that women who attend ANC have the positive influence to use the PPFP service and counseling about PPFP during ANC should be strengthened.

Women who had counseling about PPFP during PNC visit were more likely to use FP compared to women who did not get counseling about PPFP. The finding is in agreement with studies conducted in Gondar and Aksum towns [18,19]. This implies that counseling about PPFP during PNC can prevail women to utilize the FP service. Women whose menses resumed after giving birth were 1.75 times as likely to use PPFP, compared to women whose menses did not resume during the study period. The finding was in agreement with studies conducted in Kenya, Gondar town, and Aksum town [18,19,23]. This is likely because women will be more aware of fertility returning when their menses resume and they will be prompted to use FP.

Despite the high level of institutional delivery and PNC reported in the study, only around half (48.5% and 48.3%,) of the women had counseling on PPFP during their delivery and PNC, respectively. This implies that there is missed opportunity as the delivery and PNC services are the two crucial points whereby most women are supposed to get counseling on PPFP.

## Limitations of the study

The study revealed relevant findings that have paramount significance for the PPFP strategies and programs. Still, there were few limitations we needed to take into account. Generalization to Addis Ababa City has to be cautious since the participants who came to the health facilities might be different from women in other communities and because of the degree of sampling error. Of course, all the sub-cities were included in the study to make it as representative as possible, but sampling variance occurs because of recruiting a single health center from each sub-city. In addition, there could be recall bias on certain variables, like counseling during ANC or delivery of PNC because of the time gap between the study period and the specific services. Lack of a standard validated tool to assess knowledge and attitude was one of the challenges we faced in this study.

## Conclusion and recommendations

Postpartum family planning use in Addis Ababa city was encouraging. Still three out of the ten postpartum women were not using any form of PPFP which could possibly put them at risk of having unintended or closely-spaced pregnancy. Levels of counseling on PPFP during their institutional delivery and postnatal care was quite unsatisfactory. Having previous FP information, having FP information from a health facility visit, follow-up for antenatal care, counseling about FP during postnatal care, menses resumption after delivery, and commencing sexual activity after delivery were associated with increased PPFP use. The health system in the City and the healthcare providers should work on reaching any woman who is not accessing the PPFP and antenatal care services, creating awareness on PPFP, and improving counseling services on PPFP during delivery services and postnatal care. Further study has to be conducted using mixed method (qualitative and more rigorous quantitative study designs) to have in-depth information on the knowledge, attitudes, and determinants of PPFP use.

**Annex 1:** Table of sample size calculation using single population proportion formula.

Objectives	Expected proportion	Design effect	Non response rate	Estimated sample size	Reference
Objective 1: Knowledge Level	Taking p = 68.2% proportion of good knowledge on PPFP	1.5	10%	550	[15]
Objective 2: Attitude Level	Taking p = 51% proportion of favourable attitude on PPFP	1.5	10%	633	[15]
Objective 3: PPFP Use	Taking p = 80.3% proportion PPFP use	1.5	10%	401	[20]

PPFP: Postpartum family planning.

**Annex 2:** Table of sample size calculation by using two-population proportion formula.

Variable	Proportion in exposed (P_1_)	Proportion in un exposed (P_2)_	Sample size in exposed (n_1_)	Sample size in un exposed (n_2_)	n = n_1+_n_2_	Design effect	Non response rate	Final sample size	Reference
Educational status of the women	22%	2.3%	43	43	86	1.5	10%	142	[15]
Menses resumption after delivery	41%	59%	121	121	242	1.5	10%	399	[20]
Attending ANC	21%	1.3%	40	40	80	1.5	10%	132	[15]
FP counselling during PNC	67%	33%	34	34	68	1.5	10%	112	[19]

ANC: Antenatal care, FP: Family planning, PNC: Postnatal care.

## Supporting information

S1 File English questionnaire. (DOCX)

S2 File Amharic questionnaire. (DOCX)

S1 Data Research dataset. (SAV)
