# Supplementary material for: Family planning utilization and associated factors among postpartum women in Addis Ababa, Ethiopia, 2018
Source: PLoS One. 2021 Jan 22;16(1):e0245123. doi: 10.1371/journal.pone.0245123 (PMC7822255; doi:10.1371/journal.pone.0245123)
Supplement: S1 File — (DOCX) [file pone.0245123.s001.docx]

## Information Sheet, Consent Form and Questionnaire English Version

Hello Ms My name is --------------------- am here on behalf of Lema Tafa student of **Santé Medical college** school of public health. We are conducting a study on Knowledge, attitude, use and associated factors of Family planning among post partum women attending child immunization clinic in this health center. You are invited to take part in a research study. Before you decide whether to participate, you need to understand, why the research is being done and what it would involve. Please take the time to listen as I read the following information. Please ask me if there is anything that is not clear, or if you would like more information. When all of your questions have been answered and you feel that you understand this study, you will be asked if you wish to participate in the study. Purpose of the Study and Study Requirements the study entitled “*Knowledge, Attitude, Use and associated factors of Family planning among post partum women attending child immunization clinic* in Addis Ababa, Ethiopia”. According to the research eligibility, criteria you are selected as one of study participant by chance as you are attending child immunization clinic from selected health center. The study will help us to understand KAP of contraceptive and associated factors so that intervention can be done to increase contraceptive coverage. If you agree to take part in the study, you will also be asked to answer questions in relation to your socio -demography, KAP of contraceptive and reason you are not using contraceptive. This will take you about 20 minutes.
**Risks**- An inconvenience may be the time and effort you take to be a participant. You may find one or more questions that we ask to be upsetting or emotionally sensitive. You do not have to respond to any question that makes you uncomfortable.

**Benefits**- There is no direct benefits to you for participating in the study. You may find an indirect benefit in knowing you have participated in an important study that could help others in the future.

**Confidentiality**- The information that is collected during the interview will be kept privacy. No one will be told that you have participated in the study. The study team will make every effort to protect your privacy and maintain the confidentiality of all the information that you provide. Your name or other identifiers will not be included in reports from this study. Data will be stored in a computer and locked box dedicated to this study that only the study team can access. We will not share any of your information with your parents.

**Voluntariness**- Your participation in this study is voluntary. If you decide not to participate, you will not lose any existing benefits to which you are entitled. If you agree to participate in this study, you may end your participation at any time without penalty or loss of existing benefits to which you are entitled. If you decide to take part, you are free to skip any questions. You are free to withdraw at any time without affecting your relationship with the service providers. If you have any questions on the study, you can contact **Lema Tafa** by using this address: Cell phone: 0913277380.

Informed Consent

You have been already briefly informed about the study and clearly understand the objective.
Now please tell me would you be willing to participate in the study? 1. Yes 2. No
1. Yes, Thanks! Conduct the interview
2. No, Thanks! Proceed to the next eligible participant

Date of interview (Ethiopian calendar) _____/_____/____ Started time ---------- end time-------

Name of health Centre: ____________________________
Name of interviewer______________________ Signature__________

Section -1 Socio -demographic characteristics of the participants

| Q.no | Questionnaires | Response | Skip |
| --- | --- | --- | --- |
| 101 | How old are you? | ……… |  |
| 102 | Have you ever attended school? | 1. Yes 2. No | If no skip to Q. 104 |
| 103 | What is your educational level? | 1. Primary  2. Secondary  3. Diploma  4. Degree and above |  |
| 104 | What is your religion? | 1. Orthodox  2. Muslim  3. Protestant  4. Others |  |
| 105 | What is your marital status? | 1. Married  2. Single  3. Divorced  4. Widowed |  |
| 106 | What is your current occupation? | 1. Self employee  2. Government employee  3. House wife  4. Others |  |
| 107 | What is educational level of your husband? | 1. No formal education  2. Primary  3. Secondary  4. Diploma  5. degree and above |  |
| 108 | What is your husband occupation? | 1. Unemployed  2. Daily laborer  3. Private employee  4. Government employee  5. Merchant  6. Others……… |  |

Section -2 Source of contraceptive methods and knowledge of the post partum women

| Q.no | Questions | Response | | Skip |
| --- | --- | --- | --- | --- |
| 201 | Have you heard of any contraceptive methods? | 1. Yes 2. No | |  |
| 202 | What is source of information about FP?  ( Multiple response is recommended ) | 1. Health facility  2. Mass media (TV, Radio, etc…)  3. Health extension workers  4. friends  5. family  6. Others (specify)………….. | |  |
| 203 | Which FP do you know?  (Multiple response is recommended ) | 1. Oral pill  2. Emergency pill  3. Condoms  4. IUD  5. Implant  6. Injection  7.Sterilization (female and male)  8. LAM  9. Calendar method  10. Standard day method  11. With drawl method  12. Others | |  |
| 205 | From where someone can obtain the FP method?  (Multiple response is recommended ) | 1. Public health facility  2. Private health facility/clinic  3. Pharmacy/Drug shop  4. Others (specify) ………… | |  |
| **Knowledge measuring questionnaire of women towards PPFP** | | | | |
| 206 | PPFP helps to prevent unwanted pregnancies | | 1. Yes 0. No |  |
| 207 | PPFP helps to prevent possible maternal death and illness | | 1. Yes 0. No |  |
| 208 | PPFP helps to limiting number of children | | 1. Yes 0. No |  |
| 209 | PPFP helps to space child | | 1. Yes 0. No |  |
| 210 | Fertility resumed after stopping contraceptive | | 1. Yes 0. No |  |
| 211 | Exclusive breast-feeding used as FP | | 1. Yes 0. No |  |
| 212 | woman can start contraception before the menstruation begins after delivery | | 1. Yes 0. No |  |

Section -3 Attitude of post partum women towards Contraception

| Q.no | Questionnaires | Response |
| --- | --- | --- |
| 301 | Husband decide if wife wants to use family planning | 1. Strongly agree  2. Agree  3. Neutral  4. Disagree  5. Strongly disagree |
| 302 | PPFP is good for mother and child health | 1. Strongly disagree  2. Disagree  3. Neutral  4. Agree  5. Strongly agree |
| 303 | Discussing PPFP use with partner is good | 1. Strongly disagree  2. Disagree  3. Neutral  4. Agree  5. Strongly agree |
| 304 | Using contraceptive can cause infertility | 1. Strongly agree  2. Agree  3. Neutral  4. Disagree  5. Strongly disagree |
| 305 | Men should share the responsibility of family planning use. | 1. Strongly disagree  2. Disagree  3. Neutral  4. Agree  5. Strongly agree |
| 306 | Post partum contraceptive helps a mother to regain her strength before her next baby | 1. Strongly disagree  2. Disagree  3. Neutral  4. Agree  5. Strongly agree |
| 307 | Encourage your friends to use PPFP | 1. Strongly disagree  2. Disagree  3. Neutral  4. Agree  5. Strongly agree |
| 308 | Un married women can use contraceptive | 1. Strongly disagree  2. Disagree  3. Neutral  4. Agree  5. Strongly agree |
| 309 | Using contraceptive could affect cultures | 1. Strongly dis agree  2. Disagree  3. Neutral  4. Agree  5. Strongly agree |
| 310 | Religion forbids contraceptive | 1. Strongly dis agree  2. Disagree  3. Neutral  4. Agree  5. Strongly agree |

Section -4 ፡Family Planning use in the postpartum period.

| Q.no | Questionnaires | Response |
| --- | --- | --- |
| 401 | Did you use a FP method within 12 months after delivery? | 1. Yes  2. No |
| 402 | If yes to 401, what method did you use?  (Circle All Mentioned By Respondent) | 1. Pill  2. IUD  3 .Injectable  4 .Male Condom  5. Female condom  6. Implant  7. Others(specify)-------------- |
| 403 | From where did you get the FP method? | 1. Government health facility 2. Private health facility 3. NGO facility 4. Pharmacies/drug venders |
| 404 | When did you start using the method after delivery? | 1. ------------------weeks after birth/Months |
| 405 | If you were not used FP method, in the first 12 months why didn’t use?  (Circle All Mentioned By Respondent) | 1. Fear of side effects 2. Want to deliver soon 3. Fear of change in breast milk by FP methods 4. no knowledge about FP  5. Spousal not present near/No sexual contact  6. Feeling of not susceptible to Pregnancy due to breastfeeding 7. Feeling of not at risk of pregnancy due to amenorrhea 8. Absence of FP methods in my residence 9. Lack of money for FP service 10. Absence of chosen method 11. Others, specify------------- |
| 406 | Currently do you use FP method | 1. Yes 2. No |
| 407 | If yes for Q. 406, which method of family planning you use? | 1. Pill 2. IUD 3. Inject able 4. Implant 5. Others (specify) …………………………… |

Section -5 Health services characteristics of post partum women

| Q.no | Questionnaires | Response | Skip |
| --- | --- | --- | --- |
| 501 | Did you attend ANC clinic for your recent child | 1. Yes 2. No |  |
| 502 | If yes for Q. 501 How many times did you attend the ANC clinic | 1. One visit 2. Two visit 3. Three visit 4. Four and above visit |  |
| 503 | Did you counseled for FP during ANC visit | 1. Yes 2. No |  |
| 504 | Where did you delivered your recent child | 1. Hospital 2. Health centre 3. Home |  |
| 505 | Did you counseled for FP during delivery | 1. Yes  2. No |  |
| 506 | Did you visited health facility after giving recent birth | 1. Yes 2. No |  |
| 507 | Reason for visiting health facility after giving birth | 1. For immunization 2. For post natal care 3. To get treatment 4. For family planning 5. Others specify………. |  |
| 508 | When you counseled for Family planning | 1. For immunization 2. For post natal care 3. To get treatment 4. For family planning |  |

Section – 6 Reproductive history and Perceived risk of pregnancy of post partum women

| Q.no | Questionnaires | Response | Skip |
| --- | --- | --- | --- |
| 601 | How many pregnancy do you have | 1. 1  2. 2  3. 3  4. 4 and above |  |
| 602 | How many number of children do you have | 1. 1 2. 2 3. 3 4. 4 and above |  |
| 603 | How long after you were delivered recent one | 1. Less than 3 months 2. 4-6 months 3. 7-9 months 4. 10-12 months |  |
| 604 | What is the gaps of birth intervals between recent and previous birth | 1. First birth 2. < 2 years   3. 2-3 years  4. > 3 years |  |
| 605 | Your menses resumed after recent child | 1. Yes 2. No | If no skip |
| 606 | What is the month your menses resumed after your recent child | 1. < 3 months 2. 4-6 months 3. 7-9 months 4. Others (specify)   …………….. |  |
| 607 | Have you started sexual intercourse after recent birth | 1. Yes 2. No |  |
| 608 | What is the weeks of sexual intercourse you were started | 1. < 6 weeks 2. 6 weeks-3 months 3. 4-6 months 4. 7-9 months |  |
| 609 | Have you started contraceptive currently | 1. Yes 2. No |  |
| 610 | If yes for Q.609 ,When you started | 1. Immediate after birth 2. 6 weeks-3 months 3. 4-6 months 4. 7 month and above |  |
