# Supplementary material for: Family planning utilization and associated factors among postpartum women in Addis Ababa, Ethiopia, 2018
Source: PLoS One. 2021 Jan 22;16(1):e0245123. doi: 10.1371/journal.pone.0245123 (PMC7822255; doi:10.1371/journal.pone.0245123)
Supplement: S2 File — (DOCX) [file pone.0245123.s002.docx]

## Information Sheet, Consent Form and Questionnaire Amharic Version

በድህረ ወሊድ ግዜ ውስጥ ላሉት እናቶች የተዘጋጀ ቃለ መጠይቅ

**መግቢያ እና የፈቃደኝነት ስምምነት ቅፅ**

ጤና ይስጥልኝ እኔ ስሜ ………………………… ይባላል፡፡ በ ሳንቴ ሜድካል ኮሌጅ የህብረተሰብ ጤና ትምህርት ክፍል የመጨረሻ አመት ተማሪ የሆነው፤ ለማ ጣፋ የእናቶች ከወሊድ በኃላ የቤተሰብ ምጣኔ አገልግሎት እዉቀት፤ አመለካከት ፤አጠቀቃም እና እንዳይጠቀሙ የሚያደርጉት ችግሮች በሚል ምርምር በማካሄድ ላይ ናቸው፡፡ ጥናቱ ለኢትዮጽያ መንግስትና አጋር ድርጅቶች እንዲሁም በማህበረሰቡ ውስጥ ለሚገኙ የቤተሰብ ምጣኔ አገልግሎት መሪዎች ውሣኔ ሰጪነትይጠቅማል ተብሎ የተዘጋጀ ነው፡፡ እርሶ በዚህ ጥናት እንዲሳተፉ በዚህ ክፍል ውስጥ ካሉት እናቶች በእድል ተመሣሣይ፡በጥናቱ ጠቃሚ የተባሉትን መረጃዎች ለማግኘት የሚከተሉትንና ተመሣሣይ ጥያቄዎችን አቀርብሎታለሁ (ስለ አለፈው ወሊድ፤ ስለ ቤተሠብ ምጣኔ አገልግሎት አጠቃላይ ስለ ቅድመ ወሊድ ክትትል፤ስለ ቤተሰብ ምጣኔ እዉቀት፤አመለካከት፤መጠቀም እና እንደይጠቀሙ የምያደረጋቸዉ ሚክናቶች) ይሆናል ፡፡ ይህ ቃል መጠይቅ 20 ደቂቃ ያክልይወስዳል፡፡

አስጊ ሆነታዎች -ከጥያቄዎቹ መካከል ለመመለስ የማይፈልጉት ካለ ሊነግሩን ይችላሉ እንዲሁምበማንኛውም ሰአት ያለምንም ቅድመ ሁኔታ ቃለ መጠይቁን ማቆም ይችላሉ፡፡

ጥቅም- ጥናቱ ውስጥ በመሳተፎት ቀጥተኛ ጥቅም አያገኙም፤

ሚስጥር ጠባቂነት-በዚህ ቃለመጠይቅ የተገኙ ማናቸውንም መረጃዎች ከጥናቱ ባልደረባዎቸ ውጪ በፍፁም ለማንምየማይገለፅ መሆናቸውንና በሪፖርቱም ላይ የተሣታፊዎች ስም የማይገለፅ መሆኑን እና መረጃዎችን ለዚህ ጥናት አገልግሎት ከሚውል ሳጥን ውስጥ እና በኮምፒውተር ውስጥ በትንቃቄ የሚቀመጡ መሆናቸውን ከወዲሁ ለማስረዳት እንፈልጋለን፡፡

ፍቃድኝነት- በጥናቱ ውስጥ ለመሰተፍ የእርሶ ፍቃደኛ ከሆኑ ብቻ ነው፡፡በጥናቱ ላይ ማንሣት የሚፈልጋቸውን ማናቸውንም ጥያቄ ካለዎት በሚከተለው አድራሻ ለ ለማ ጣፋ ብለው በስልክ ቁጥር 0913277380 መደወል ይችላሉ፡፡

ቃለ መጠይቁን መጀመር እንችላለን? 1. አዎ 2. አልችልም

1. አዎ ….. …. አመሰግናለዉ ቃለመጠየቁን መቀጠል
2. አልችልም …… አመሰግናለዉ ወደ ቀጣይ መለፍ
   ቀን ………….የጀመሩበት ሰአት ………የጨረሱበት ሰአት …………

የተቋሙ ስም ………………..….ጤናጣቢያ ቃለ መጠያቁን የሞላ ስም ናፍርማ…………

| ተ.ቁ | ጥያቄዎች | መልሶች | ወደሚቀጥለው ጥይቄ ይሂዱ |
| --- | --- | --- | --- |
| 101 | እድሜዎ ስንት ነው፡፡ | ___________ |  |
| 102 | መደበኛ ትምህርት ቤት ገብተው ያውቃሉ? | 1. አዎ  2. አይ | መልሱ 2 ከሆነ ወደ ጥያቄ 104 ይሂዱ |
| 103 | የትምህር ደረጃዎት? | 1. 1ኛ ደረጃ 2. 2ኛ ደረጃ 3. ዲፕሎማ 4. ድግሪ እና ከዚያ በላይ |  |
| 104 | ሀይማኖት | 1. ኦርቶዶክስ 2. ሙስሊም 3. ፕሮቴስታንት 4. ሌላ………… |  |
| 105 | የጋብቻ ሁኔታ | 1. ያገባ 2. ያላገባ 3. የፈታ 4. በሞት የተለየ |  |
| 106 | የሥራ ሁኔታ | 1.የግል ስራ  2. የመንግስት ሥራ  3. የቤት እመቤት  4. ሌላ ………………. |  |
| 107 | የባለቤተዎ የትምህርት ደረጃ | 1. ያልተማረ 3. 2ኛ ደረጃ 2. የመጀመሪያ ደረጃ 4. ዲፕሎማ   5. ድግሪና ከዚያ በላይ |  |
| 108 | የባለቤተዎ የስራ ሁኔታ | 1.ሥራ የሌለው 4.የመንግስት ሰራተኛ  2. የቀን ሰራተኛ 5. ነጋዴ  3. የግል ሠራተኛ 6. ሌላ … |  |

ክፍል -1፡ማህበራዊ እና ስነ ህዝብ መረጃ

ክፍል-2፡በድህረወሊድያሉ ሴቶችላይስለወሊድ መቆጣጠሪያያላቸውን ዕውቀትለማወቅየተዘጋጀ ቃለ መጠይቅ

| ተ.ቁ | | ጥያቄዎች | መልሶች | | ወደሚቀጥለውጥይጥያቄ ሂድ | |
| --- | --- | --- | --- | --- | --- | --- |
| 201 | | ስለ ወሊድ መቆጣጠሪያ ሰምቶ ያውቃሉ ? | 1. አዎ 2. አይ | | (መልስዎት 2 ከሆነወደ ጥያቄ ቁጥር 204 ይሂዱ) | |
| 202 | | ስለ ወሊድ መቆጣጠሪያ ያገኙት ከየት ነው ? | 1. ጤናተቋም 2. ከሚዲያ (ቴለተቭዥን ፣ራድዮወዘተ…) 3. ከጤና ኤክስቴንሽን ሰራተኞች 4. ከጓደኞች 5. ከቤተሰብ 6. ከሌላ……… | |  | |
| 203 | | የትኛውን የቤተሰብ ምጣኔ አይነት ያውቃሉ (በተጠቀሰው ቁጥር ላይ ያክብቡ ) ? | 1. በአፍ የሚዋጥ ክኒን  2. የድንገተኛ  3. ኮንደም  4. በማህፀን ውስጥ የሚቀመጥ (ሉፕ)  5. በክንድ ስር የሚቀመጥ (ኢንፕላንት  6. መርፌ  7. ቋሚ የእርግዝና መከላከያ(ሴት/ወንድ)  8. ጡት በመጥበት  9. ከለንደር በመጠቀም  10. በየ ወሩ ቀን በመቁጠረ  11. ከግንኙነት ቦሃላ የወንድ ዘረ ከመጻን ውጭ መፍሰስ  12. ሌላ ከለ…………. | |  | |
| 205 | | የቤተሰብ ምጣኔ አንድት ሴት የት ሊያገኝ ይችላል | 1. ከህብረተሰብ ጤና ጠቋም 2. ከግል ጤና ተቋም (ክሊንክ ) 3. ከመድኃኒት መሽጫ   4. ከሌላ …………… | |  | |
| ከ ወሊድ ቦሃላ ስለ ቤተሰብ ምጠኔ የእናቶችን እዉቀት የሚለካ ትያቄዎች | | | | | | |
| 206 | ከወሊድ ቦሃላቤተሰብ ምጠኔ መጠቀም ያልተፈለገ ዕርግዝናን ይከላክላል | | | 1.አዎ 0. አይ | |  |
| 207 | ከወሊድ ቦሃላቤተሰብ ምጠኔ መጠቀም ከእናት ሞትና ህመም ይከላክላል | | | 1.አዎ 0. አይ | |  |
| 208 | ከወሊድ ቦሃላቤተሰብ ምጠኔ መጠቀም ሊኖር የሚገባ ልጅን ለመወሰን ይጠቅመል | | | 1አዎ 0. አይ | |  |
| 209 | ከወሊድ ቦሃላቤተሰብ ምጠኔ መጠቀም ልጆችን አራርቆ ለመዉለድ ይጠቅመል | | | 1.አዎ 0. አይ | |  |
| 210 | የወሊድ መቆጣጠሪያ ካቆሙ እርግዝና ይፈጠራል | | | 1.አዎ 0. አይ | |  |
| 211 | የእናት ጡት ማጥባት ብቻ እንደ ቤተሰብ ምጣኔ መንገድ ያገለግላል | | | 1. አዎ 0. አይ | |  |
| 212 | አንዲት ሴት ከ ወለደች ቦሐላ የወርአበባዋ ከመምጣቱ በፊት የወሊድ መቆጣጠሪያመጀመር ትችላለች | | | 1. አዎ 0. አይ | |  |

ክፍል -3: በድህር ወሊድ ያሉ ሴቶች ላይ ካለወሊድ መቆጣጠሪያ ያላቸውን አመለካከት ለማወቅ የተዘጋጀ መጠይቅ

| ተ.ቁ | ጥያቄዎች | መልሶች |
| --- | --- | --- |
| 301 | ሚስት የቤተሰብ ምጣኔ መጠቀም ብትፈልግ ባል ምርጫዋ ላይ ሊወስን ይገባል ? | 1. በጣም እስማማለሁ 2. እስማማለሁ 3. አለውቅም 4. አልስማማም 5. በጣም አልስማማም |
| 302 | ስለ ደህር ወሊድ የቤተሰብ ምጣኔ ለእናትና ልጅ ጤና አስፈላጊ /ጥሩ /ነው ? | 1. በጣም አልስማማም 2. አልስማማም 3. አለውቅም 4. እስማማለሁ 5. በጣም እስማማለሁ |
| 303 | ስለ ደህር ወሊድ የቤተሰብ ምጣኔ ከትዳር አጋረው ጋር መወያየት ጥሩ ነው ? | 1. በጣም አልስማማም 2. አልስማማም 3. አለውቅም 4. እስማማለሁ 5. በጣም እስማማለሁ |
| 304 | የወሊድ መቆጣጠሪያ መጠቀም መካንነት ያመጣል? | 1. በጣም እስማማለሁ  2. እስማማለሁ  3. አለውቅም   1. አልስማማም 2. በጣም አልስማማም |
| 305 | ወንዶች የቤትሰብ ምጣኔ መጠቀም ሃላፊነታቸውን መውሰድ አለባቸው ? | 1. በጣም አልስማማም 2. አልስማማም 3. አለውቅም 4. እስማማለሁ 5. በጣም እስማማለሁ |
| 306 | ደህር ወሊድ የወሊድ መቆጣጠሪያ ለእናት ቀጣይ እርግዝናዋ ጥንካሬ ይሰጣታል ? | 1. በጣም አልስማማም 2. አልስማማም 3. አለውቅም 4. እስማማለሁ 5. በጣም እስማማለሁ |
| 307 | የቤተሰብ እቅድ እንድጣቀሙ ጓደኞችሽን ታበረታታለሽ ? | 1. በጣም አልስማማም 2. አልስማማም 3. አለውቅም 4. እስማማለሁ 5. በጣም እስማማለሁ |
| 308 | ያላገባች ሴት የወሊድ መቆጣጠሪያ መጠቀም ትችላለች ? | 1. በጣም አልስማማም 2. አልስማማም 3. አለውቅም 4. እስማማለሁ 5. በጣም እስማማለሁ |
| 309 | የወሊድ መቆጣጠሪያ መጠቀም በህል ላይ ተፅህኖ ያደርሳል ? | 1. በጣም እስማማለሁ 2. እስማማለሁ 3. አለውቅም 4. አልስማማም 5. በጣም አልስማማም |
| 310 | ሃይማኖት የወሊድ መቆጣጠሪያ ይከለክላል ? | 1. በጣም እስማማለሁ 2. እስማማለሁ 3. አለውቅም 4. አልስማማም 5. በጣም አልስማማም |

**ክፍል -4** በድህር ወሊድ ወቅት ሴቶች የወሊድ መቆጣጠሪያ አጠቃቀማቸውን ወይም ትግበራቸውን ለማወቅ የተዘጋጀ መጠየቅ

| ተ.ቁ | ጥያቄዎች | መልሶች | ወደሚቀጥለውጥይጥያቄ ሂድ |
| --- | --- | --- | --- |
| 401 | የቤተሰብ ምጣኔ ከወሊድ በኋላ በአንድ ዓመት ውስጥ ተጠቅመው ነበር ? | 1. አዎ 2. አይ |  |
| 402 | ተራ ቁጥር 401 መልስዎት ተጠቅምያሉሁ ከሆነ ምን አይነት የወሊድ መቆጣጠሪያመንገድ ተጠቅመው ነበር ? | 1. ክኒን 2. ሉፕ 3. መርፌ 4. የወንድ ኮንደም 5. የሴት ኮንደም 6. በክንድ ስር የሚቀመጥ (ኢንፕላንት)   ሌላ ………………………… |  |
| 403 | የወሊድ መቆጣጠሪያውን የተጠቀሙት ከየት ነበር ? | 1. ከመንግስት ጤና ጠቋም 2. ከግል ጤና ተቋም (ክሊንክ ) 3. መንግስታዊ ካልሆነ ድርጅት 4. ከመድኃኒት መሽጫ |  |
| 404 | የወሊድ መቆጣጠሪያ ከወለዱ በኋላ የተጠቀሙት መቼ ነበር | ---------------------- ሳምንት/ወር |  |
| 405 | የቤተሰብ ምጣኔ ከወሊድ በአንድ ዓመት ውስጥ ያልተጠቅሙ ከሆነ ያል ተጠቀሙት ለምንድን ነበር ?( መልሶቹን መክበብ) | 1. የጎንዮሽ ጉዳቱን በመፍራት 2. መውለድ በመፈለግ በማሰብ 3. በወሊድ መቆጣሪያ ምክንያት የጡት ወተት ችግር ያመጠል ብየ 4. ስለ ቤተሰብ ምጣኔ እውቀት አልነበረኝም 5. የትዳር አጋረ ባለመኖሩ/ግንኙነት ስለ አለረኩ 6. ስለማጠባ ለእርግዝና አልጋለጥም ብዬ 7. የወር አበባ ስለማላይ ለእርግዝና አልጋለጥም ብዬ በማሰብ   8. የቤተሰብ ምጣኔ በምኖርበት አከባቢ አለመኖር 9. ለቤተሰብ ምጣኔ አገልግሎት ገንዘብ ስለሌለኝ  10. አማራጭ ስላል ነበረኝ 11. ሌላ------------------ |  |
| 406 | ማንኛውም የቤተሰብ ምጣኔ ዓይነት በአሁን እየተጠቀሙ ነው ? | 1. አዎ 2. አይ |  |
| 407 | የተራ ቁጥር 406 አዎ እጠቀማለሁ ከሆነ የትኛውን የወሊድ መቆጣጠሪያ መንገድ ይጠቀማሉ | 1. ክኒን 2. ሉፕ 3. መርፌ 4. በክንድ ስር የሚቀመጥ (ኢንፕላንት)   5. ሌላ ----------------------------- |  |

ክፍል- 5፡ በድህር ወሊድ ያሉ ሴቶች በጤና አገልግሎት ዙሪያ ያለቸውን ሁኔታ ለማወቅ የተዘጋጀመጠየቅ

| ተ.ቁ | ጥያቄዎች | መልሶች | ወደሚቀጥለውጥይጥያቄ ሂድ |
| --- | --- | --- | --- |
| 501 | የመጨረሻ ልጅዎት አርግዘው የቅድመ ወሊድ ክትትል አድርገው ነበር ? | 1. አዎ 2. አይ |  |
| 502 | በተራ ቁጥር 501 መልስዎ አዎ ከሆነ ምንያህል ጊዜ የቅድመ ወሊድ ክትትል አድረገዋል ? | 1. አንድ ጊዜ 2. ሁለት ጊዜ 3. ሶስት ጊዜ   4. አራት እና ከዚያ በላይ |  |
| 503 | በቅድመ ወሊድ ወቅት ስለ ቤተሰብ ምጣኔ የምክር አገልግሎት አግኝተዋል ? | 1. አዎ 2. አይ |  |
| 504 | የመ ጨረሻልጅዎን የወለዱት የት ነበር ? | 1. ሆስፒታል 2. ጤና ጣቢያ 3. ቤት |  |
| 505 | በወሊድ ወቅት ስለ ቤተሰብ ምጣኔ የምክር አገልግሎት አግኝተዋል ? | 1. አዎ 2. አይ |  |
| 506 | የመጨረሻልጅ ከወለዱ በኋላ ወደ ጤና ተቋም ሄደው ነበር ? | 1. አዎ 2. አይ |  |
| 507 | ከወሊድ በኋላ ወደ ጤና ተቋም የሄዱት ለምንድነበር ? | 1. ለክትባት 2. ለድህር ወሊድ ክትትል 3. ለህክምና 4. ለቤተሰብ ምጣኔ 5. ሌላ -------- |  |
| 508 | መቼ ነበር ለቤተሰብ ምጣኔ የምክር አገልግሎት ያገኙት ? | 1. በክትባት ወቅት 2. በድህረወሊድ ወቅት 3. ለህክምና በሄድኩ ወቅት 4. የቤተሰብ ምጣኔ አገልግሎት ወቅት 5. ሌላ………………… |  |

**ክፍል-6፡** በድህር ወሊድ ያሉ ሴቶች የስነተዋልዶ ታሪኮችን እና እርግዝናን ተከትሎ የሚመጡ ችግሮችን ለማወቅ የተዘጋጀመጠይቅ

| ተ.ቁ | ጥያቄዎች | መልሶች | ወደሚቀጥለውጥይጥያቄ ሂድ |
| --- | --- | --- | --- |
| 601 | ስንት ጊዜ አርግዘሻል ? | 1. አንድ ጊዝ 2. ሁለት ጊዜ 3. ሶስት ጊዜ 4. አራት እና ከዚያ በላይ |  |
| 602 | ምን ያህል ልጆች አሉሽ | 1. አንድ 2. ሁለት 3. ሶስት   4. አራት እና ከዚያ በላይ |  |
| 603 | የመጨረሻ ልጅሽን ከወለድሽ ምን ያህል ጊዜ ይሆናል? | 1. ከ3 ወር በታች 2. 4-6 ወራት 3. 7-9 ወራት 4. 10-12 ወራት |  |
| 604 | በመጨረሻ ልጅሽና በቀድሞ ልጅሽ መካከል ምን ያህል ጊዜ ልዩነት ነበር ? | 1. የመጀመሪያ እርግዝናዬ ነው 2. ከ2 ዓመት በታች 3. ከ 2-3 ዓመት 4. ከ 3 ዓመት በላይ |  |
| 605 | የወር አበባሽ የመጨረሻ ልጅ ከወለድሽ በኋላ አይተሸል? | 1. አዎ 2. አይ |  |
| 606 | የመጨረሻ ልጅሽን ከወለድሽ በኋላ የወር አበባያየሽው መቼ ነበር ? | 1. ከ3 ወር በፊት 2. 4-6 ወራት ውስጥ 3. 7-9ወራት ውስጥ 4. ሌላ ---------------- |  |
| 607 | የመጨረሻ ልጅሽ ከወለድሽ በኋላ የግብረስጋ ግንኙነት ማድረግ ጀምረሻል ? | 1. አዎ 2. አይ |  |
| 608 | የግብረስጋ ግኑኝነት ከወለድሽ በኋላ የጀመርሽው መቼ ነው ? | 1. ከ6 ሳምንት በፊት 2. ከ6 ሳምንት -3 ወራት ውስጥ 3. ከ4 -6 ወራት ውስጥ 4. ከ7 -9 ወራት ውስጥ |  |
| 609 | የወሊድ መቆጣጠሪያ አሁን መጠቀም ጀምረሻል? | 1. አዎ 2. አይ |  |
| 610 | የተራ ቁጥር 609 መልስዎ አዎ ከሆነ መቼ ጀመርሽ? | 1. ወዲያው እንደወለድሽ 2. ከ6 ሳምንት -3 ወራት ውስጥ 3. ከ4 -6 ወራት ውስጥ 4. ከ7 ወራት እና ከዚያ በላይ ያሉት ጊዜያት ውስጥ |  |
